# Maternal Inositol Status and Neural Tube Defects: A Role for the Human Yolk Sac in Embryonic Inositol Delivery?

**DOI:** 10.1093/advances/nmaa100

**Published:** 2020-09-05

**Authors:** Stephen W D'Souza, Andrew J Copp, Nicholas D E Greene, Jocelyn D Glazier

**Affiliations:** Maternal and Fetal Health Research Centre, St. Mary's Hospital, School of Medical Sciences, Faculty of Biology, Medicine and Health, Manchester Academic Health Science Centre, University of Manchester, Manchester, United Kingdom; Newlife Birth Defects Research Centre, Developmental Biology and Cancer Department, UCL Great Ormond Street Institute of Child Health, London, United Kingdom; Newlife Birth Defects Research Centre, Developmental Biology and Cancer Department, UCL Great Ormond Street Institute of Child Health, London, United Kingdom; Division of Evolution and Genomic Sciences, School of Biological Sciences, Faculty of Biology, Medicine and Health, Manchester Academic Health Science Centre, University of Manchester, Manchester, United Kingdom

**Keywords:** *myo*-inositol, folate, pregnancy, polyol, placenta, fetus

## Abstract

Supplementation with *myo*-inositol during the periconceptional period of pregnancy may ameliorate the recurrence risk of having a fetus affected by a neural tube defect (NTD; e.g., spina bifida). This could be of particular importance in providing a means for preventing NTDs that are unresponsive to folic acid. This review highlights the characteristics of inositol and describes the role of *myo*-inositol in the prevention of NTDs in rodent studies and the evidence for its efficacy in reducing NTD risk in human pregnancy. The possible reduction in NTD risk by maternal *myo*-inositol implies functional and developmentally important maternal–embryonic inositol interrelationships and also suggests that embryonic uptake of *myo*-inositol is crucial for embryonic development. The establishment of active *myo*-inositol cellular uptake mechanisms in the embryonic stages of human pregnancy, when the neural tube is closing, is likely to be an important determinant of normal development. We draw attention to the generation of materno-fetal inositol concentration gradients and relationships, and outline a transport pathway by which *myo*-inositol may be delivered to the early developing human embryo. These considerations provide novel insights into the mechanisms that may underpin inositol's ability to confer embryonic developmental benefit.

## Introduction

**FIGURE 1:**
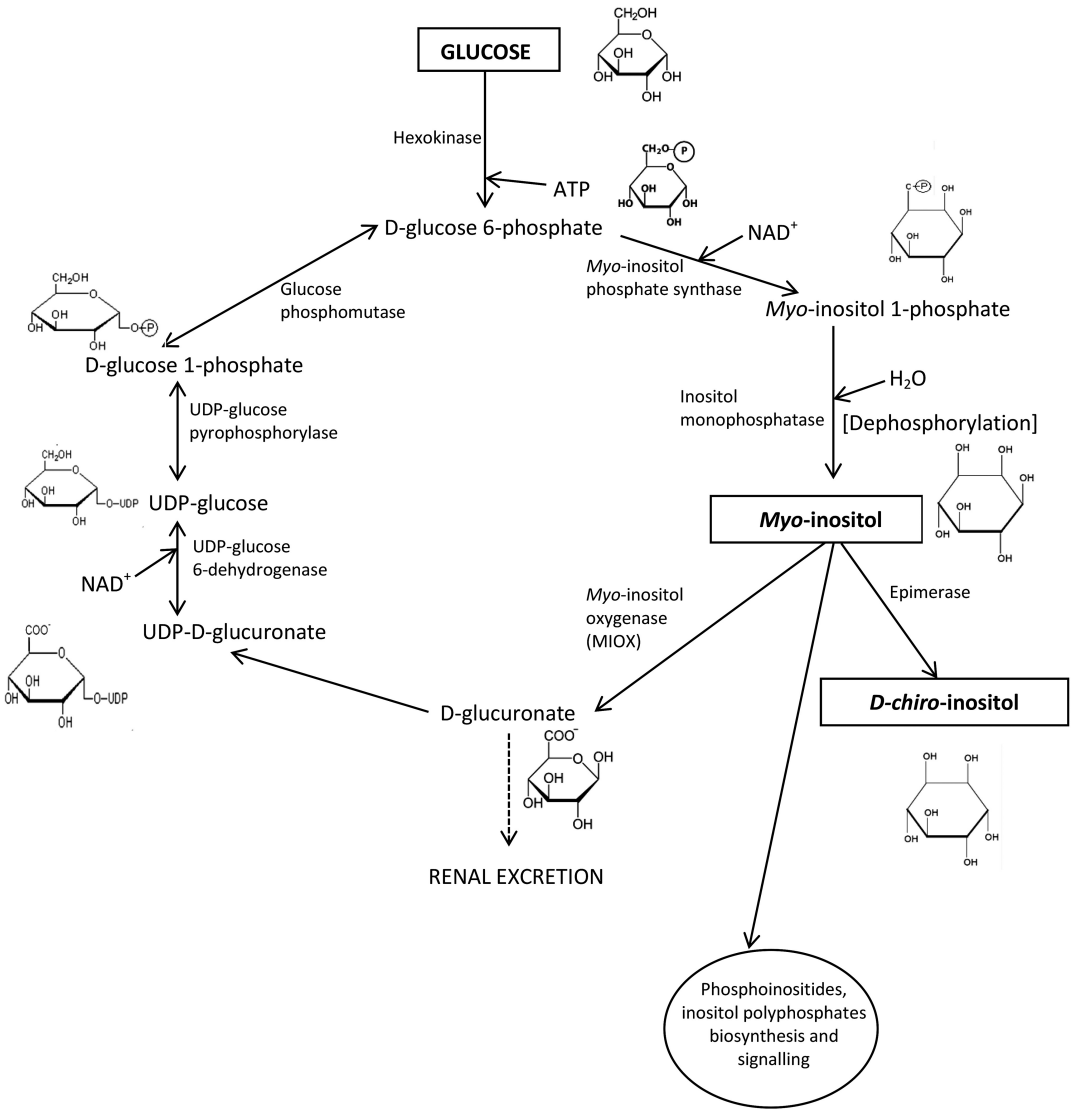
*Myo*-inositol biosynthesis and catabolism in mammalian tissues. Most tissues can synthesize *myo*-inositol following phosphorylation of d-glucose to glucose-6-phosphate with subsequent synthesis of *myo*-inositol-1-phosphate, which, following dephosphorylation, forms free *myo*-inositol. This can be incorporated into the phosphoinositides cycle, be further catabolized to d-glucuronate for renal excretion or, following a series of enzymatic reactions, be converted back to glucose-6-phosphate. Additionally, *myo*-inositol can be converted to *D-chiro*-inositol through the enzymatic action of an epimerase.

Inositol is present in a variety of foods including nuts, seeds, vegetables and fruit, with *myo*-inositol (**Figure 1**) being the predominant isomeric form (1). Inositol may exist as free *myo*-inositol, *myo*-inositol–containing phospholipids (phosphoinositides), or as phytic acid (inositol hexakisphosphate), which is hydrolyzed mostly to free inositol before absorption from the gut (1, 2). *Myo*-inositol is a sugar alcohol referred to as a “cyclitol” or “polyol” due to its cyclic structure containing 6 hydroxyl groups (Figure 1). The differing spatial orientation of its 6 hydroxyl groups gives rise to 9 stereoisomeric inositol forms (1, 3). Adults typically consume 1 g of *myo*-inositol/d (1–5). In addition, most tissues are able to produce *myo*-inositol endogenously (∼4 g/d) (1, 3) from d-glucose by de novo synthesis involving the following: *1*) phosphorylation of glucose by hexokinase to glucose 6-phosphate, *2*) conversion of glucose 6-phosphate to *myo*-inositol-1-phosphate by 1-d-myo-inositol-phosphate synthase (encoded by *ISYNA1* gene) (6, 7), and *3*) dephosphorylation of inositol-1-phosphate by inositol monophosphatase to generate free *myo*-inositol (Figure 1) (1, 4, 8).

*Myo*-inositol is the predominant inositol stereoisomer in mammalian cells (1, 3, 5, 9). The plasma concentration of *myo*-inositol in healthy adults is ∼30 μmol/L, which is thought to reflect the balance of dietary inositol intake, cellular uptake by Na^+^- and energy-dependent co-transporters distributed to several tissues (10–12), endogenous synthesis from glucose, cellular metabolism, and clearance (3, 5, 13, 14). *Myo*-inositol clearance is accompanied by catabolism of *myo*-inositol by *myo*-inositol oxygenase (MIOX) to d-glucuronate followed by renal excretion (1, 3, 8, 15) (Figure 1). *Myo*-inositol can also be converted to *D-chiro*-inositol by an epimerization catalytic step (Figure 1), although the conversion rate is relatively low (1, 4).

This review integrates current concepts regarding the importance of *my*o-inositol in embryonic development, its efficacy in reducing the risk of neural tube defects (NTDs), materno-fetal inositol relationships, and the role of inositol transporters in embryonic/fetal *myo*-inositol provision.

## Inositol, Early Embryonic Development, and Neural Tube Closure

As early as preimplantation stages of mammalian development there is a notable requirement for *myo*-inositol, with uptake increasing from the 1-cell to the blastocyst stage; *myo*-inositol incorporation into the phosphatidylinositol cycle promotes cell proliferation (16–19). Mouse embryos exposed to *myo*-inositol in vitro display an increase in proliferative activity and a faster cleavage rate resulting in more blastocysts (20, 21). Hence *myo*-inositol transporters must be active over this period of preimplantation development to meet *myo*-inositol demand if this cannot be met by endogenous production. Indeed, in the mouse preimplantation embryo, *myo*-inositol uptake appears to involve mainly Na^+^-dependent inositol transporters with a minor contribution from Na^+^-independent mechanisms (18). Early developmental induction of carrier-mediated transporters also has relevance for the progressive growth of the embryo and fetus at postimplantation stages, as neural tissues contain high concentrations of *myo*-inositol, achieved through active transport of *myo*-inositol (3).

Studies in rodent embryos have shown that inositol deficiency results in cranial NTDs in cultured embryos (22–24), indicating a crucial role for inositol in neural tube closure. Inositol supplementation is effective in reducing embryopathy, including NTDs associated with hyperglycemia, diabetes, and folate deficiency (4). Neural tube closure is particularly sensitive to inositol status in the *curly tail* mouse strain, a multigenic model in which the major gene defect is a hypomorphic allele of the grainyhead-like transcription factor 3 gene (*Grhl3*) (25). NTDs in *curly tail* mutants are unresponsive to prevention by folic acid (or methionine), but in contrast, inositol supplementation reduces the incidence of spina bifida (4, 26), an effect that can be mediated by either *myo*- or *D-chiro-*inositol. Each stereoisomer reduces the incidence of spina bifida in a dose-dependent manner, inferring that both forms of inositol can be taken up by the embryo (27). The mechanism underlying the higher preventive capacity of *D-chiro*-inositol as compared with *myo-*inositol is not currently understood (27). Stimulation of protein kinase C (PKC) activity, specifically of the β1 and γ isoforms, with a lesser contribution from PKCζ, contributes to the ameliorating effects of *myo*-inositol on impaired neural tube closure (26, 28). It has been speculated that the enhanced protective effect of *D-chiro*-inositol could be by eliciting greater downstream effects on PKC activation (27).

These observations suggest there must be a pathway available for delivery of maternal *myo*-inositol (or derived active inositol metabolites) to the developing embryo during pregnancy, when neural tube closure occurs. Defining the embryonic inositol pathway will be an important determinant for future *myo*-inositol–focused therapeutic strategies, informed by an understanding of materno-fetal *myo*-inositol interrelationships and mechanisms driving embryonic/fetal delivery.

## Materno-Fetal Inositol Relationships

Lower serum *myo*-inositol concentrations have been reported in mothers of children with spina bifida (29), suggesting a possible predisposing association as found in animal models. Whether *myo*-inositol synthesis might be altered in NTDs has not been determined. The *ISYNA1* gene is expressed in adult human tissues as well as in placenta and yolk sac (7, 30), leading to the hypothesis that genetic defects of *myo*-inositol synthase may result in low maternal and/or embryonic intracellular *myo*-inositol concentrations, predisposing to NTD pathogenesis (31). In a case-control triad study of children with spina bifida aged 1 to 3 y and their parents, genetic defects of the *ISYNA1* gene were investigated but no association with spina bifida was noted (31).

**FIGURE 2:**
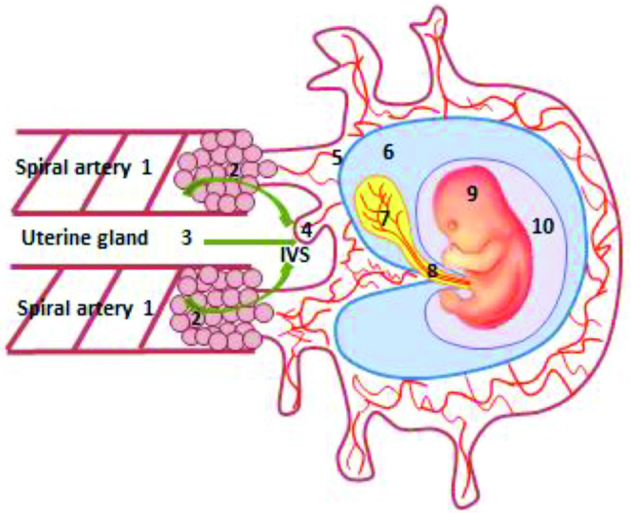
Histiotrophic pathway for embryonic nutrient delivery involving the human yolk sac. Nutrient-containing maternal plasma ultrafiltrates from spiral arteries (1) percolates through plugs of trophoblast cells (2; curved arrows) that occlude their openings, to reach the IVS, along with secretions from the uterine glands (3; straight arrow). Nutrients, including inositol, are taken up by the developing placenta (4), and pass through the villous mesenchymal core (5) with connections to the exocoelomic cavity (6) that contains coelomic fluid of high inositol concentration, which bathes the yolk sac (7). Nutrients taken up by the yolk sac from the coelomic fluid enter the vitelline circulation of the yolk sac (7) that is in direct continuity with the embryonic circulation, or else pass from the yolk sac cavity along the direct connection of the vitelline duct to the embryonic gut (8) of the embryo (9), which is contained within the amniotic cavity (10). Reproduced with permission from Nyree Sardena, University of Manchester. IVS, intervillous space.

A higher fetal inositol concentration relative to maternal exists in several species (32). Indeed, in the early weeks of human pregnancy (5–12 wk), inositol concentration in the embryonic compartment is already significantly higher than in maternal serum (33), suggesting that active, carrier-mediated transport mechanisms for inositol are established early in pregnancy and/or there is substantial placental/fetal production of inositol. In matched samples of human intervillous, coelomic, and amniotic fluids, taken over this gestational period, the concentration of inositol in each fluid was significantly higher than that in maternal serum (33). The concentration of inositol in the coelomic fluid that bathes the yolk sac within the exocoelomic cavity (**Figure 2**) was particularly high, reported to be ∼10-fold higher than maternal serum (33). The high *myo*-inositol tissue concentrations in the developing central nervous system (CNS), skeletal, and cardiac muscle (34) emphasize the high embryonic demand for this nutrient. *Myo*-inositol, along with other polyols, may have multiple functions during embryogenesis, acting as osmolytes to promote expansion of the amniotic and coelomic cavities, providing precursors of cell membrane components and supplying substrates for the pentose phosphate pathway necessary for the synthesis of nucleic acids (33). Moreover, the high concentration of polyols, including sorbitol, in the embryonic compartment may reflect an early dependence on polyol metabolic pathways that serve to maintain ATP concentrations and redox potential while the embryo develops in a low oxygen environment (33).

The high early pregnancy demand for *myo*-inositol is also reflected in rodent pregnancy, as suggested by the decline in total fetal inositol content as pregnancy advances (35). This phenomenon may also be related to altered placental de novo synthesis of *myo*-inositol, as evidenced by the decline in human pregnancy of umbilical venous concentration between midgestation and term (36). By midgestation of human pregnancy, umbilical venous *myo*-inositol concentration is higher than maternal (∼6-fold) (36), and later, in newborn infants at term, umbilical venous *myo*-inositol concentration is ∼4-fold higher than in maternal blood (37, 38). Neonatal *myo*-inositol concentrations gradually decline over the first few months after birth to attain concentrations comparable to adults (38).

Fetal production of *myo*-inositol is also likely based on the evidence that umbilical arterial blood returning to the placenta has a significantly higher *myo*-inositol concentration than that in umbilical venous blood (36, 37), generating a negative umbilical venous-arterial concentration difference for *myo*-inositol that is not seen for other polyols (37). Staat et al. (39) infused stable isotope–labeled inositol into mothers 2 h before delivery and demonstrated that the plasma enrichment ratio of fetal (cord)/maternal tracer concentration deviated markedly from the value of 1, interpreted as inositol in the fetal circulation not being directly dependent on maternal inositol concentration. Although a higher fetal inositol concentration was achieved, this did not appear to be all maternally derived, with a fetal/maternal isotopic enrichment ratio in the umbilical vein of 0.12, suggesting that only ∼12% of fetal inositol originated from maternal plasma. However, the 2-h period of this study was considered to be a potential limitation, such that dilution by placenta-derived inositol (36, 37) is also a possibility.

## Inositol Transporters

Although mammalian serum concentrations of *myo*-inositol are ∼30–70 μM, cellular concentrations can reach as high as 30 mM (40). This capacity for cellular *myo*-inositol accumulation is mediated by carrier-mediated transporters. *Myo*-inositol is transported by 3 different transporters: the lower affinity H^+^-coupled inositol transporter (HMIT) (41), and two higher affinity Na^+^-dependent *myo*-inositol transporters (SMIT), called SMIT1 (42, 43) and SMIT2 (44), members of the solute carrier *SLC5* gene family of Na^+^-dependent glucose co-transporters (45). SMIT1 and SMIT2 react to osmotic imbalances (46–48), whereas no major osmoregulatory function has been reported for HMIT. Despite their common inositol transporting ability, it is worth noting that these transporters do not share amino acid sequence homology (41, 49).

### HMIT

Activity of HMIT, encoded by *SLC2A13*, is stimulated by an inwardly directed H^+^-gradient through an increase in maximal velocity (V_max_) with no change in substrate affinity (K_m_ ∼100 μM for *myo*-inositol) (41). HMIT is predominantly expressed in the brain, with greatest abundance in the hippocampus, hypothalamus, cerebellum, and brain stem, although it is also expressed in other tissues such as the kidney (41, 50). The HMIT protein shows the same membrane topology as other *SLC2* glucose transporter (GLUT) family members, and hence its designation as GLUT13, comprising 12 transmembrane domains, an enlarged loop between the sixth and seventh transmembrane domain, and N- and C-termini located within the cytoplasm. In addition, it possesses an extracellular loop between the ninth and tenth transmembrane domains (41, 49, 50). HMIT is generally localized to intracytoplasmic membranes (50), and in neurons is located to the Golgi apparatus (51) and plasma membrane (41). *Scyllo-*, *D-chiro-*, and *muco*-inositol are also accepted as substrates, but glucose, fructose, and other hexoses are not (41). HMIT activity can, however, be reduced by common GLUT inhibitors such as phlorizin and phloretin (41). Recently, it has been proposed that HMIT mediates the transport of inositol triphosphate (IP3) and, in doing so, may play a role in the intracellular regulation of phosphoinositide signaling (51).

### SMIT1

The Na^+^/*myo*-inositol co-transporter SMIT1, encoded by *SLC5A3* (42), has a role in cellular adaptation to hypertonicity (46, 47) by accumulating *myo*-inositol, particularly in the kidney and brain (49). It has a similar tissue distribution pattern to SMIT2, being expressed in the kidney, heart, skeletal muscle, placenta, and brain, although, in the CNS, SMIT1 has a relatively high expression (49, 52, 53). The preferred substrate for SMIT1 is *myo*-inositol (K_m_ = 55 μM) and its transport of fucose allows its activity to be distinguished from that of SMIT2 (49). The human *SLC5A3* gene has been proposed to give rise to mRNA splice variants: one that encodes the SMIT1 protein containing 14 transmembrane helices and two other variants SMIT1-2 and SMIT1-3 that encode SMIT1 isoforms with different C-termini but without the last transmembrane domain (49).

Mice homozygous null for *Slc5a3* exhibit a 77% reduction in *myo*-inositol content by embryonic day (E) 10.5, which is still evident at late fetal stages, with an 84% reduction reported at E18.5 (35). No histological defects of the placenta or a variety of other fetal tissues were noted (35). Offspring are reported as having a normal weight and gross appearance at birth (35), while other reports state that newborns have a small body size, short limbs, drooped forelimbs and skull, and a curved spine as compared with wild-type mice (54). Notwithstanding these discrepancies, a consistent observation is that *Slc5a3*-null mice die shortly after birth, apparently due to neurological dysfunction, cyanosis, and hypoventilation (35, 55). Despite the severely diminished *myo*-inositol content of the fetal forebrain of *Slc5a3* homozygous null mutants (35), brain phosphoinositide concentrations were not altered in the fetus (56, 57).

Interestingly, the survival and viability of *Slc5a3*-null fetuses could be rescued by enriching drinking water of pregnant dams with 1% (54, 55, 57) or 2% (58) *myo*-inositol. Supplemental maternal *myo*-inositol intake between days E9.5 and E15.5 in mice was found to be a crucial gestational window for phenotypic rescue (55). However, surviving *myo*-inositol–supplemented *Slc5a3* knockout offspring still exhibited *myo*-inositol depletion in the brain, kidney, and skeletal muscle. This was associated with severe abnormalities of the peripheral nerves—in particular, the sciatic nerve—and bones with delayed prenatal skeletal mineralization, diminished postnatal bone density, and altered bone remodeling (55, 57, 58). Together, these phenomena confirm the importance of SMIT1 transporter activity during fetal development and its crucial contribution to fetal *myo*-inositol accretion, but also infer that compensatory SMIT1-independent mechanisms are able to prevent neonatal lethality and restore offspring viability following maternal inositol supplementation. However, these activities failed to fully restore fetal or offspring tissue *myo*-inositol concentrations in surviving mutants.

### SMIT2

SMIT2 is encoded by *SLC5A11* (also called *KST1*) and is 43% identical in nucleotide sequence to SMIT1 (44). SMIT2 is expressed in human heart, skeletal muscle, kidney, liver, and placenta, and more weakly in the brain (59). SMIT2 transport of *myo*-inositol is Na^+^-dependent with a K_m_ of 120 μM. SMIT2 is inhibited by phlorizin and exhibits stereospecific cotransport of both d-glucose and d-xylose but does not transport fucose (which is transported by SMIT1) (49). SMIT2 also transports *D-chiro*-inositol (K_m_= 130 μM), with similar affinity to *myo*-inositol. In contrast, *D-chiro*-inositol is not transported by SMIT1, allowing distinction between the two transporters (49). The physiological substrate of SMIT2 is postulated to be *myo*-inositol as plasma *D-chiro*-inositol concentrations are low (49). In humans, a lower serum *myo*-inositol concentration was found in mothers with the *SLC5A11* CC genotype compared with the TT genotype, although there was no association between this genetic polymorphism and spina bifida (31).

Schneider (49) draws attention to the co-expression of SMIT1 and SMIT2 in several tissues, which may influence the kinetics of inositol transport depending on their relative contributions (44). In the *curly tail* (folic acid–unresponsive) mouse, *D-chiro*-inositol is more effective than *myo*-inositol in the prevention of NTDs (27), suggesting that SMIT2-mediated inositol uptake activity is pivotal in eliciting such downstream preventive responses. In clinical studies, the efficacy of *D-chiro*-inositol in combination with *myo*-inositol has been reported in the treatment of polycystic ovarian syndrome (60) and gestational diabetes (61). *D-chiro*- and *myo-*inositol could be beneficial based on the relatively high *myo*-inositol concentrations reported in reproductive organs compared with blood (62) and the insulin-sensitizing properties of these inositol stereoisomers in experimental models of hyperglycemia and diabetes (1).

Together, these studies highlight the importance of *myo*-inositol provision during gestational development and implicate the existence of maternal–fetal inositol transfer pathways that confer embryonic developmental benefits, potentially including NTD prevention. This concept is strengthened by the following collective observations:

1. *Myo*-inositol supplementation prevents the reduced *myo*-inositol content and NTDs induced in cultured rat conceptuses by hyperglycemia, suggesting that *myo*-inositol depletion is involved in diabetic embryopathy (63, 64). Tissue *myo*-inositol concentration of fetuses with a NTD phenotype was more profoundly diminished than in unaffected fetuses of diabetic rat dams. Additionally, reduced yolk sac inositol tissue concentration in the diabetic group was restored to control concentrations with maternal inositol supplementation (65).
2. An inositol-sensitive developmental pathway is suggested from inositol supplementation studies showing reduced incidence of NTDs in mouse models (24).
3. *Myo*-inositol uptake into mouse blastocysts is mediated by a Na^+^-dependent, temperature-sensitive mechanism, that can be inhibited by raised glucose concentrations (13), suggesting an importance for *myo*-inositol as early as the blastocyst stage (16).
4. The high inositol concentration in coelomic fluid in direct contact with the human yolk sac (Figure 2), during early pregnancy and organogenesis phases of embryonic development, is likely to contribute to the maintenance of inositol metabolic pathways that promote optimal embryonic growth and development (33).

## A Role for the Placenta and Yolk Sac in Embryonic/Fetal Inositol Delivery?

As well as supporting evidence for the existence of functional carrier-mediated *myo*-inositol uptake mechanisms in early embryonic development, additional studies draw attention to the potential capacity of the placenta and yolk sac as a site of *myo*-inositol uptake and/or *myo*-inositol synthesis. In early human pregnancy, when the neural tube and other embryonic organ systems are forming, blood flow through the utero-placental circulation has yet to be initiated. Maternal blood flow to the maternal spiral artery openings of the placenta is initially occluded by plugs of migrating endovascular trophoblast cells (66, 67) (Figure 2). The functional advantage of this arrangement is that blood flow is minimized so that the embryo can undergo organogenesis in a low oxygen environment, which may limit the potential of any damaging effect of reactive oxygen species (67, 68). Hence, the spiral artery openings remain plugged over most of the duration of the first trimester (when embryonic organogenesis is occurring) and it is the diffuse network of intercellular spaces forged between the amassed endovascular trophoblast cells that provides the direct link between the arterial lumen of the spiral arteries and the intervillous space surrounding the chorionic (placental) villi (69–71). The intervillous space contains a colorless fluid thought to consist of a mixture of maternal plasma that has percolated through the network of intercellular channels between the trophoblast cells, and carbohydrate- and lipid-rich secretions (“histiotroph”) from the maternal uterine glands that transit directly to the intervillous space (66, 67) (Figure 2). Nutrients contained therein are proposed to be taken up by the epithelial syncytiotrophoblast layer of the placental villi (66, 67, 72, 73). Towards the end of the first trimester, at ∼10 wk of gestation, the trophoblast cell “plugs” start to loosen, allowing the maternal arterial circulation to flow to the intervillous space with the ensuing full establishment of the uteroplacental circulation in concert with contemporaneous elaboration of the feto-placental circulation (67, 71).

Hence, during critical periods of human development, transfer of nutrients and precursors for embryonic development and growth occurs prior to the onset of established blood flow through the placental circulation. This poses a very interesting question: How is delivery of maternal nutrients, including *myo*-inositol, that are essential for embryonic development and growth, achieved over this very vulnerable period of developmental embryogenesis when the neural tube is closing? Although not well defined, the current proposition is that maternal–embryonic nutrient transfer is mediated by the mode of nutrition known as “histiotrophic” nutrition (74), as illustrated in Figure 2. In this scheme, the yolk sac is likely to have a crucial role in the provision of embryonic nutrition (74) as it is *1*) connected directly to the developing embryo by the vitelline circulation (71, 74) and *2*) connected by the vitelline duct to the embryonic gut (75). The latter anatomical connection remains patent until ∼8–9 wk of fetal life when it is obliterated (75). Because the yolk sac exists only to ∼12 wk of human pregnancy (76), it has remained elusive from investigation. In this context, the rodent visceral yolk sac, which persists throughout pregnancy and is readily accessible as an outer membrane that envelops the fetus, becomes a useful surrogate for study, particularly as it known that the yolk sacs of both species express common transport moieties and receptors, such as folate receptor α, cubilin, and megalin (77–80). Additionally, the rodent yolk sac is well known to contribute to embryonic nutrition (81, 82).

At term, the human placenta expresses the *SLC5A3* and *SLC5A11* genes encoding SMIT1 (53) and SMIT2 (59) *myo*-inositol transporters, respectively. However, expression in early pregnancy has not been examined. In a preliminary study, we investigated the expression of inositol transporters in pooled cDNA from 30 first-trimester placentas using gene-specific primers to each of the three *myo*-inositol transporters and confirmed the expression of the *SLC5A3* and *SLC5A11* genes. Additionally, we detected the placental expression of *SLC2A13* transcripts encoding HMIT, showing that all three genes are expressed in first-trimester placenta (unpublished data; N Sardena and J Glazier, 2018). We hypothesized that, within the setting of a high inositol concentration in coelomic fluid that is in direct contact with the yolk sac's outer surface (33), the human yolk sac would also express these three inositol transporters, in common with the placenta. To test this hypothesis, we examined *SLC2A13, SLC5A3*, and *SLC5A11* gene expression in human yolk sacs, matched to first-trimester placentas from the same pregnancies. The expression of all three inositol transporter genes was confirmed in human yolk sac (unpublished data; N Sardena and J Glazier, 2018). This is in partial agreement with recent analysis of the human yolk sac transcriptome (30), which also identified *SLC2A13* (HMIT) and *SLC5A11* (SMIT2) transcripts, although the expression of *SLC5A3* (SMIT1) transcripts was not reported in that study (30).

Hence, our findings identify for the first time the co-expression of all three inositol transporters in the first-trimester human placenta and pregnancy-matched yolk sacs. This raises the possibility that these three transporters may be involved in carrier-mediated uptake of *myo*-inositol and play a role in embryonic inositol provision and developmental pathways dependent on *myo*-inositol availability. Further functional studies are required to test this postulate and to characterize the pathways involved that might underpin the delivery of maternally derived *myo*-inositol to the developing embryo at critical developmental time points. It is possible, by analogy to other cell types that HMIT- and SMIT-mediated activities may each contribute to cellular *myo*-inositol uptake under different prevailing conditions.

It is rather difficult to reconcile the concepts that maternally derived *myo-*inositol does not impact on embryo/fetal inositol, when maternal *myo*-inositol supplementation confers embryological benefits, unless maternal *myo*-inositol drives downstream metabolic signaling events that have a direct influence. Hence, an improved understanding of cellular *myo*-inositol transport and uptake mechanisms provides a mechanistic framework to delineate how inositol might exert beneficial effects.

## Inositol and Pleiotropic Effects

In the E10.5 rat conceptus (10–12 somites), cellular uptake of *myo*-inositol and accumulation into the tissue pool and lipid components were inhibited competitively by increasing concentrations of d-glucose, an effect not observed with other hexoses (83). In diabetic mothers, cellular depletion of *myo*-inositol might therefore contribute to the associated increased risk of birth defects, including NTDs (84). Indeed, a variety of models of diabetic complications exhibit intracellular depletion of *myo*-inositol (85). Further, there is evidence supporting a possible role of *myo*-inositol supplementation in reducing the incidence of diabetic embryopathy, including NTDs (4, 86–88). These outcomes have stimulated interest in the pleiotropic effects of inositol with regard to the following: *1*) the insulin-mimetic effects of *myo*-inositol (1), *2*) generation of inositol phosphoglycan signaling molecules from glycosylphosphatidylinositol membrane anchoring moieties (89), *3*) the role of intracellular *myo*-inositol as an organic osmolyte (90, 91), and *4*) involvement in signaling pathways through generation of IP3, phosphatidylinositol phosphate lipids [phosphatidylinositol biphosphate (PIP2)/ phosphatidylinositol triphosphate (PIP3)], and other derivatives (92–95). These multiple physiological actions of inositol have led to several clinical studies investigating whether *myo*-inositol or *D-chiro-*inositol may be effective in prophylaxis and treatment of diabetes or gestational diabetes (96, 97), and have also initiated assessment of whether maternal *myo*-inositol supplementation in the periconceptional period might be effective in reducing NTD risk (4, 85).

## Folate, Inositol, and NTDs—Evidence for Folate-Sensitive and Inositol-Sensitive Etiologies

By the fourth week postconception, the embryonic neural plate has undergone a series of coordinated morphological events culminating in the formation of the closed neural tube (98, 99). Failure of the processes underpinning neural tube closure leads to NTDs, including anencephaly and spina bifida (98–100). These are among the most serious congenital malformations of the CNS, with a prevalence worldwide of 0.5–2.0 per 1000 established pregnancies (101) and 0.9 per 1000 births in Europe (102–104). Prenatal screening has led to early detection and termination of NTD-affected pregnancies, resulting in lower NTD-affected birth rates in some countries (105, 106). Women with ≥1 previous pregnancies associated with NTDs have a higher risk of a subsequent affected pregnancy (107). The recurrence risk after a single affected pregnancy of 3.2% increases to 11.8% after two affected pregnancies (107). Patterns of inheritance indicate a major genetic contribution to NTD risk (99, 108, 109). Yet, it is also well recognized that maternal nutrition has an important role in influencing NTD prevalence, with interplay between genetic variants and nutritional factors contributing to NTD risk (99).

This has led to a sustained interest in elucidating how maternal nutritional intake may impact NTD risk. Given the evidence that the risk of having a NTD-affected pregnancy is increased by low maternal blood folate concentrations (99, 110–112), focus has centered on the effects of maternal B vitamins such as folate and vitamin B-12, with one-carbon metabolism and methylation postulated to be cellular loci that can be modulated by these nutrients to influence NTD risk (100, 113).

However, although an inverse relation between maternal folate concentration and NTD risk prevails (114, 115), the range of maternal blood folate concentrations determined in affected cases rarely indicates clinical folate deficiency (99). Moreover, the intake of folic acid supplements used preconception reduces the risk of NTD occurrence and recurrence but does not prevent all cases (116). A randomized trial (116) reported that the 3.6% of NTD recurrence with no folic acid fell to 0.6% with folic acid supplements. The efficacy of folic acid has been replicated in a series of further intervention trials (112), with compelling evidence that maternal intake of folic acid supplements in the periconceptional period significantly reduces NTD risk (117), with prevention also achieved by folic acid fortification of flour (118). Clinical case studies have therefore drawn attention to a subset of NTDs (∼30%) referred to be as “folic acid resistant” NTDs (4, 119, 120) that occur despite the use of folic acid in the periconceptional period.

Research in the *curly tail* (*Grhl3*) mouse mutant demonstrated a preventive effect of inositol on NTDs that are resistant to folic acid supplementation (4, 26). Further, a lower maternal *myo*-inositol concentration in humans is associated with a greater risk of having a spina bifida–affected infant (29). Such observations led to the use of *myo*-inositol in a highly selected group of women to prevent NTD recurrence in a subsequent pregnancy (119–121). A cohort of 12 women with a history of folic acid–resistant NTDs, who were counseled to take a combination of *myo*-inositol and folic acid supplements before a subsequent pregnancy, gave birth to healthy infants without NTDs (119–121). While the number of study participants was too small to draw firm conclusions, the findings encouraged the view that *myo*-inositol may contribute towards NTD prevention, advocating the need for a more fully powered study (4).

Progressing this concept, a pilot double-blind clinical trial assessed whether the use of inositol supplements in combination with folic acid would be more protective than folic acid alone (122). The PONTI (Prevention of Neural Tube defects by Inositol) study recruited women who wished to become pregnant after experiencing ≥1 previous NTD-affected pregnancy (119, 122). Supplementation of 14 pregnancies with *myo*-inositol (1 g) and folic acid (5 mg) daily, starting before conception and continuing until the end of 12th week of pregnancy, led to the birth of healthy unaffected infants in all cases. By comparison, 19 established pregnancies supplemented with a placebo and folic acid resulted in the birth of 18 normal infants and 1 fetus diagnosed with anencephaly. Importantly, no adverse effects of the *myo*-inositol supplementation in mothers or their infants were reported (122).

Although, collectively, these maternal *myo*-inositol supplementation studies (119–122) have yielded encouraging results, they are as yet of insufficient size to support the combined use of *myo*-inositol and folic acid supplements as a public health intervention to prevent NTDs. A larger number of women would need to be recruited to a fully powered study (4, 122). However, taken together, they do suggest that *myo*-inositol, as a simple and inexpensive nutrient supplement, could potentially be protective against NTD recurrence in folic acid-nonresponsive cases, making it an appealing candidate for testing in future clinical studies. Furthermore, they raise the possibility that *myo*-inositol could modulate the causative underlying defect(s) that leads to the formation of an NTD, or enhance the process required for neural tube closure that is not responsive to folic acid supplementation alone. The loci of inositol's action remain to be fully delineated, although impaired phosphoinositide and inositol polyphosphate signaling has been proposed as a potential pathway in NTD pathogenesis (4).

## Conclusions

*Myo*-inositol, in addition to folic acid, is a nutritional supplement that may abrogate the pathogenesis of NTDs. *Myo*-inositol can be synthesized endogenously from glucose-6-phosphate and, together with inositol from the diet, may influence circulating concentrations. In the first trimester of pregnancy, the coelomic fluid that bathes the yolk sac, an organ of maternal–embryonic nutrient exchange, contains high concentrations of inositol and other polyols. These likely derive from maternal plasma that percolates from the maternal spiral arteries and/or from uterine decidual gland secretions, together with potential synthesis by the placenta and embryo/fetus. This indicates that the polyol metabolic pathway in the human conceptus is highly active in the early weeks of pregnancy to maintain ATP concentrations and cellular redox potential while the embryo develops in a low oxygen environment. We report preliminary evidence that the *SLC2A13* (HMIT), *SLC5A3* (SMIT1), and *SLC5A11* (SMIT2) genes are all expressed in both first-trimester placenta and human yolk sac matched from the same pregnancy. Together, these transporters may mediate *myo*-inositol uptake from coelomic fluid. *Myo*-inositol synthesis de novo from glucose-6-phosphate may also be possible as the *ISYNA1* gene, which encodes *myo*-inositol-1-phosphate synthase, is expressed in the human placenta and yolk sac. These lines of evidence lead us to hypothesize a functional role for the human yolk sac in *myo*-inositol provision for the developing embryo, by uptake from coelomic fluid, as well as capacity for de novo inositol synthesis.

Animal studies indicate that *myo*-inositol supplementation reduces the frequency of NTDs in a mouse strain that is nonresponsive to folic acid. Moreover, diabetic rats treated with *myo*-inositol had fewer NTD-affected fetuses. In clinical studies, *myo*-inositol supplements may have a similar effect in preventing NTDs unresponsive to folic acid, lending support to the importance of maternal *myo*-inositol for the developing embryo. This article highlights the possibility that the human yolk sac may be a contributor to embryonic *myo*-inositol provision, underscoring the potential for *myo*-inositol to participate in multiple inositol signaling pathways that regulate a diverse array of cellular mechanisms. Thus, further studies investigating the expression and functionality of human yolk sac *myo*-inositol transporters, as well as *myo*-inositol synthesis capacity, would lead to an improved appreciation of the role of the yolk sac in embryonic development, when neural tube closure and embryonic organogenesis are occurring. Further, a better understanding of maternal *myo*-inositol transport by the human yolk sac to the developing embryo would add to the mechanistic framework that supports *myo*-inositol's use in clinical practice to prevent NTD recurrence in cases deemed folate nonresponsive.

## Notes

Human embryonic and fetal material was provided by the Joint MRC/Wellcome Trust (grant MR/R006237/1) Human Developmental Biology Resource (http://hdbr.org). Research on inositol and neural tube defects by AJC and NDEG is funded by Action Medical Research (GN2656) and Sparks and supported by the National Institute for Health Research Biomedical Research Centre at Great Ormond Street Hospital for Children NHS Foundation Trust and University College London.

Author disclosures: The authors report no conflicts of interest.

The funders did not play a role in the design, implementation, analysis, or interpretation of data.

Abbreviations used: CNS, central nervous system; E, embryonic day; HMIT, H^+^-coupled *myo*-inositol transporter; GLUT, glucose transporter; IP3, inositol triphosphate; NTD, neural tube defect; PIP2, phosphatidylinositol biphosphate; PIP3, phosphatidylinositol triphosphate; PKC, protein kinase C; SLC, solute carrier group of membrane transport proteins; SMIT, Na^+^-dependent *myo*-inositol transporter.
